# Fortified Milk Supplementation Improves Vitamin D Status, Grip Strength, and Maintains Bone Density in Chinese Premenopausal Women Living in Malaysia

**DOI:** 10.1089/biores.2018.0027

**Published:** 2019-03-01

**Authors:** Marlena C. Kruger, Yoke Mun Chan, ChinChin Lau, Lee Ting Lau, Yit Siew Chin, Barbara Kuhn-Sherlock, Linda M. Schollum, Joanne M. Todd

**Affiliations:** ^1^ School of Health Sciences, College of Health, Massey University, Palmerston North, New Zealand.; ^2^ Department of Nutrition and Dietetics, Faculty of Medicine and Health Sciences, Universiti Putra Malaysia, Serdang, Malaysia.; ^3^ Malaysian Research Institute on Ageing, Universiti Putra Malaysia, Serdang, Malaysia.; ^4^ Research Centre of Excellence for Nutrition and Non-Communicable Diseases, Universiti Putra Malaysia, Serdang, Malaysia.; ^5^ Fonterra Cooperative Ltd., Auckland, New Zealand.; ^6^ Fonterra Research and Development Centre, Palmerston North, New Zealand.; ^7^ Liggins Institute, University of Auckland, Auckland, New Zealand.

**Keywords:** body composition, bone density, Chinese women, fortified milk, grip strength

## Abstract

This study compared the effects of a high-calcium vitamin D fortified milk with added FOS-Inulin versus regular milk on serum parathyroid hormone (PTH), vitamin D status, grip strength (GS), as well as bone density in Chinese premenopausal women over 52 weeks. Premenopausal women (*n* = 133), mean age 41 (±5.1) years were randomized into control (*n* = 66; regular milk at 500 mg calcium per day) or intervention (Int; *n* = 67; fortified milk at 1200 mg calcium, 15 μg vitamin D, and 4 g FOS-Inulin per day) groups. Assessments were at baseline, weeks 12, 24, 36, and 52 for changes in vitamin D status, levels of PTH, and GS. Bone mineral densities (BMDs) of the lumbar spine (LS), femoral neck (FN), and whole body (WB) were assessed at baseline and week 52 using GE Lunar iDEXA (GE Healthcare, Madison, WI). At baseline, WB lean mass was positively associated with LS BMD (*r* = 0.30, *p* < 0.001) and FN BMD (*r* = 0.33, *p* = 0.003). Baseline 25(OH) vitamin D3 levels were 48.6 and 53.2 nmol/L (*p* = 0.57), respectively, and after the 12 months at 60.8 nmol/L (Int) versus 55.0 nmol/L (controls; *p* < 0.05 for change from baseline for both groups; no difference between groups at week 52). PTH levels decreased in both groups compared to baseline (*p* < 0.001), with no significant difference between groups. WB bone mineral content (BMC) and FN Z-score increased significantly in the Int group (*p* = 0.024 and *p* = 0.008). GS was positively associated with body weight, increasing in both groups over 52 weeks. Fortified milk improved vitamin D status, WB BMC, and Z-score of the FN, while regular milk maintained BMD. In addition, vitamin D status and GS improved.

## Introduction

In Asia, osteoporosis is rapidly increasing among women with the subsequent increasing risk of hip fracture.^1^ Low calcium intake and insufficient vitamin D status have been reported as being risk factors for low bone mineral content, risk of hip fracture, and osteoporosis among Asian women.^2–4^ Adequate consumption of calcium, protein, and a sufficient vitamin D status contribute to accretion of peak bone mass, maintenance of this bone mass, and its structural integrity throughout life, as well as positively affecting skeletal muscle mass and strength.^5,6^

Among younger adult women, Picard et al.^7^ showed that calcium intake in early adulthood influenced bone mass in premenopausal women, while Baran et al.^8^ reported a reduction in vertebral bone loss in 20 premenopausal Caucasian women aged 30- to 42-year-old with addition of dairy products to their diets over 3 years, and Daniele et al.^9^ showed that dietary calcium and vitamin D supplementation reduced total bone mineral density (BMD) loss in perimenopausal women (45^+^ years old) in Italy. In addition, a cross-sectional study of women from early childhood to young adulthood showed an association between levels of calcium/dairy intake and BMD.^10^

There have been few studies on milk or calcium supplementation and bone health in premenopausal Asian women. Ho et al.^11^ measured the percentage change in bone BMD and BMC of the spine and hip in adolescent Chinese girls aged between 14 and 16 years over 1 year of supplementation with calcium fortified soy milk. There was a significant increase in total hip BMD as well as BMC over the year but no significant changes in lumbar spine (LS) BMD and BMC. A study of 843 Chinese women aged 35–75 years in 5 rural counties of China showed a positive association between dietary calcium obtained from dairy products and radius BMD and BMC once adjusted for age and body weight.^2^

Only one study where young premenopausal Chinese women were supplemented could be found and in this study milk supplementation was shown to be of benefit, increasing bone mass over 6 months, with some effects on markers of bone resorption.^12^ The daily calcium requirement for Chinese women was suggested to be 900 mg/day to maintain balance in postmenopausal Chinese women, but the exact requirement for younger women is not known. The current reference nutrient intake for pre- and postmenopausal women in Malaysia is 1000 and 1200 mg, respectively, regardless of ethnicities. Sufficient dietary calcium is essential in young women to attain peak bone mass which could delay the development of osteoporosis in later life.^13^ In the presence of insufficient dietary calcium, absorption can be improved by consumption of a prebiotic such as oligofructose-enriched inulin (FOS-inulin). Studies by Van Der Heuvel et al.,^14^ Griffin et al.,^15^ and by Abrams et al.^16^ indicated that prebiotics could affect and improve peak bone mass accrual and help optimize peak bone mass.

The purpose of the current study was to compare the effect of a 12-month intervention with a newly formulated high-calcium milk drink with added vitamin D and FOS-Inulin in comparison with regular milk, on serum parathyroid hormone (PTH), vitamin D status, as well as bone density in premenopausal Chinese women in Malaysia. In addition, the impact on body composition and muscle strength was assessed.

## Methods

The study was approved by the Ethics Committee for Research Involving Human Subjects, Universiti Putra Malaysia.

### Study population, inclusion, and exclusion criteria

Women were recruited aged between 30 and 50 years and with a body mass index (BMI) between 16.1 and 32.1 kg/m^2^. Exclusion criteria included a history of metabolic bone disease; normal liver/kidney function; diagnosed diabetes mellitus or insulin resistance; lactose intolerance/milk allergy; regular use of calcium and/or vitamin D supplements; regular use of anti-acids containing calcium; more than two units of alcohol per day; smoking; regular use of medication that may influence bone mass; rheumatoid arthritis/autoimmune disease; and fractures in the last 6 months.

### Randomization criteria, procedures, and intervention

#### Recruitment

**FIG. 1.**
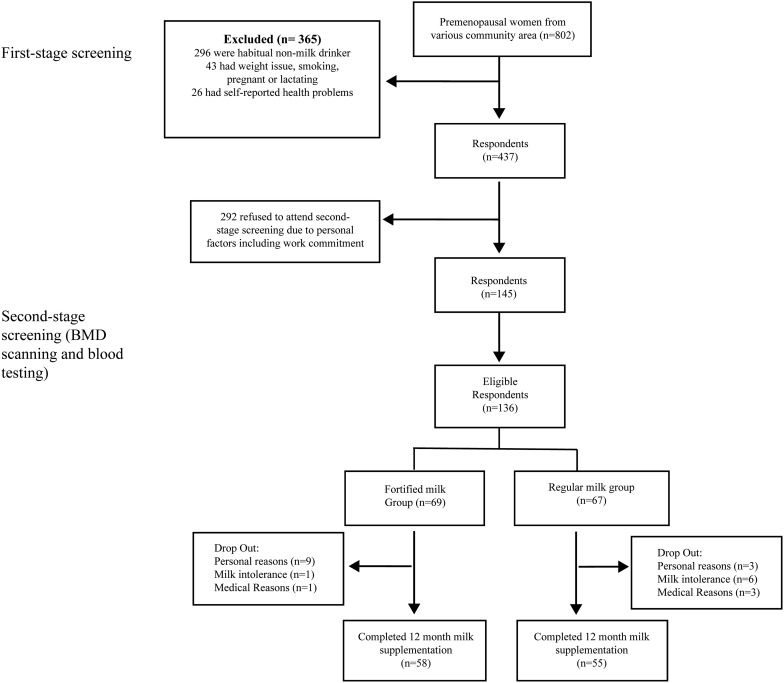
Consort diagram.

A total of 802 premenopausal community-based women were screened for eligibility using a structured questionnaire, and finger-prick cholesterol and glucose tests were done. From these, 650 women passed the initial screening exercise and agreed to a second stage screening (Fig. 1).

#### Screening visit

A screening questionnaire on general health was administered at an initial visit. A blood sample was taken between 8 and 10 am, after an overnight fast, and hematology, blood minerals, and metabolic markers were assessed. If the blood test results were normal, study subjects were asked to have BMD of femoral neck (FN) and spine (L1–L4) as well as body composition measurements (using a GE Lunar iDXA; GE Healthcare, Madison, WI). Body weight was measured to the nearest 0.1 kg using SECA scales and standing height was measured to the nearest 0.1 cm using a stadiometer. Waist and hip circumference were measured to the nearest 0.1 cm using a nonstretchable measuring tape. The medical history of each subject was recorded at this visit.

### Intervention

One hundred and thirty-six premenopausal Malaysian Chinese women were recruited with written informed consent for the trial. Of these, 133 women were included in the final analyses. The women were randomized into 2 groups, with 66 assigned to the Control group, and 67 assigned to the intervention (Int) group. The Control group received two servings per day of regular milk (500 mg calcium) and the Int group received two servings per day of fortified milk (1000 mg calcium plus 15 μg vitamin D and 4 g FOS-Inulin; Anlene^™^, Fonterra Brands, Singapore Pte Ltd.) for 52 weeks (Table 1).

**Table 1.** Composition of the High-Calcium Vitamin D FOS-Inulin Fortified Milk Powder and the Control Milk Powder, Composition Given per Day

Content/serve	Control	Intervention^a^
Fat	6.5	0.8
Calcium (mg)	428	1000
Vitamin D (μg)	0	15
FOS-Inulin (g)	0	4

^a^ Anlene^™^; Fonterra Brands (Singapore) Pte Ltd.

#### Baseline

##### Blood sampling

Blood samples were taken between 8 and 10 am (after an overnight fast) for the baseline measurements. Samples were taken to measure serum calcium, magnesium, phosphorus, lipid profile, glucose, insulin, PTH, 25(OH) vitamin D3, and C-telopeptide of type I collagen (CTx-1). The samples for measuring minerals and metabolic health were collected, processed, and analyzed immediately by local diagnostic laboratories. Plasma samples for PTH, 25(OH) vitamin D3, and CTx-1 were snap frozen and stored at −80°C and analyzed by Canterbury Health Laboratories, Christchurch, New Zealand.

##### Anthropometry

Methodology as per screening visit.

##### Questionnaires completed at baseline

A 3-day diet record, food frequency questionnaire, demographics, and a list of all medication taken in the last 6 months were recorded at baseline.

##### Follow-up measurements

At weeks 12, 24, 36, and 52, blood samples were taken between 8 and 10 am (after an overnight fast) for 25(OH) vitamin D3, PTH, and CTx-1. In addition, blood samples at weeks 12, 24, and 52 were analyzed for blood minerals, lipid profile, and glucose. Collection and analysis of the samples were conducted as per the baseline visit. Anthropometry, bone density measurements (DXA), and questionnaires were completed as at baseline. Retrospectively, a number of DXA scans were of insufficient quality/consistency to be analyzed, resulting in low final numbers for DXA data.

### Blood and bone marker analyses

PTH and CTx-1 were analyzed by electrochemiluminescence immunoassay using the Roche COBAS^®^ e411 system (Roche Diagnostics, Indianapolis, IN). 25(OH) vitamin D3 was analyzed using isotope-dilution liquid chromatography–tandem mass spectrometry.

### Compliance

Milk powder was dispensed to each subject at baseline and on a monthly basis thereafter. In addition, phone calls were made to monitor milk consumption of the subjects. Each subject was provided with a monthly diary and asked to record their milk powder intake each day.

### Statistical analyses

Based on published data, the within subject standard deviation for the primary outcome variable CTx-1 was estimated to be 0.045 ng/mL, and a representative mean CTx-1 value was taken to be 0.23 ng/mL. The corresponding standard deviation of the treatment difference was calculated as 0.064 ng/mL.

To detect a difference of 20% (i.e., 0.046 ng/mL) with a power of 90% and an alpha of 5%, we required 42 subjects per group. To allow for dropouts, and/or potentially a lower difference (16.5%), the number of volunteers required was increased to 60 per group.

SAS (SAS Institute, Inc., Cary, NC) was used for statistical analysis. Mixed models approach of repeated measures was used and the reported *p*-values for the effects of treatment group, time, and their interaction were based on a compound symmetry covariance pattern model (except for DXA data, where the unstructured covariance pattern model provided a better fit). Data were analyzed as (1) raw data, (2) difference from baseline, and (3) percentage change from baseline. For (2) and (3), the baseline results (week 0) were included in the model as a covariate and the repeated measures analysis was based on the results from during the intervention (i.e., weeks 4, 12, 24, 36 and 52). Analysis of variance was followed by *post hoc* comparisons of treatment means using the Tukey–Kramer test. Data were log_10_ transformed if required to achieve homogeneity of variance. Measurements were considered to be significantly different if *p* < 0.05.

## Results

### Baseline characteristics

The population characteristics of the premenopausal women are shown in Table 2. The mean ages for these groups were 41 and 42 years with a mean BMI of 22–23 for the Control and Int groups, respectively. The mean LS BMD was normal for both the Control (T score = 0.80) and Int (T scores = 0.84) groups. FN BMD was 0.89 and 0.87 g/cm^2^ with T scores of −0.18 and −0.39 for the Control and Int groups. Mean dietary calcium intakes were 469 mg/day for both groups. The mean 25(OH) vitamin D3 levels were 48.6 and 53.2 nmol/L for the Control versus Int groups. There were no significant differences between the groups at baseline.

**Table 2.** Baseline Characteristics of the Women

	Control (*n* = 66)	Int (*n* = 67)	SD	*p*
Age (years)	41	42	5.1	0.48
BMI (kg/m^2^)	22.3	23.0	3.44	0.86
LS BMD (g/cm^2^)	1.21	1.21	0.128	>0.99
LS T-score	0.80	0.84	1.069	>0.99
LS Z-score	0.67	0.63	1.094	0.99
Femoral neck BMD (g/cm^2^)	0.89	0.87	0.112	0.77
Femoral neck T–score	−0.18	−0.39	0.937	0.77
Femoral neck Z-score	0.01	−0.31	0.906	0.41
Calcium intake (mg)	469	469	237.5	>0.99
25(OH)vitamin D3 (nmol/L)	48.6	53.2	16.13	0.57
Grip strength (kg)	20.2	20.3	5.59	>0.99

Values are given as means and SD.

BMI, body mass index; BMD, bone mineral density; LS, lumbar spine; SD, standard deviation.

### Impact of intervention

#### General health and blood minerals

General measures of health, including fasting blood glucose and fasting lipid profiles as well as blood minerals, were within normal ranges over the trial period of 52 weeks (Table 3) with no differences between groups over time.

**Table 3.** General Health Measures and Blood Minerals over the 52 Weeks of Intervention

	Control			Int		
	Week 0	Week 24	Week 52	Week 0	Week 24	Week 52
Calcium-corr (mmol/L)	2.29 (0.010)	2.31 (0.009)	2.34 (0.009)	2.35 (0.009)	2.34 (0.009)	2.35 (0.009)
Magnesium (mmol/L)	0.95 (0.009)	0.94 (0.010)	0.94 (0.010)	0.93 (0.010)	0.91 (0.010)	0.90 (0.010)
Phosphorus (mmol/L)	1.11 (0.024)	1.10 (0.022)	1.09 (0.023)	1.13 (0.023)	1.13 (0.023)	1.11 (0.023)
Total cholesterol (mmol/L)	5.00 (0.096)	4.92 (0.097)	5.04 (0.097)	4.98 (0.099)	4.97 (0.099)	5.05 (0.099)
HDL (mmol/L)	1.64 (0.043)	1.59 (0.043)	1.58 (0.043)	1.60 (0.043)	1.66 (0.043)	1.60 (0.043)
Fasting blood glucose (mmol/L)	4.75 (0.052)	4.75 (0.053)	4.81 (0.053)	4.84 (0.054)	4.76 (0.054)	4.85 (0.054)

Values are given as least squares means (SEM). *n* = 42–60 per group.

HDL, high-density lipoprotein; SEM, standard error of the mean.

#### Vitamin D status

For assessment of the vitamin D status of the women, the Institute of Medicine (IOM) Guidelines were used where a level of >50 nmol/L is considered adequate, and <50 nmol/L is considered insufficient.^17^ A total of 58% of the Control group had 25(OH) vitamin D3 levels that were insufficient at baseline (data not shown), with 45% insufficient at baseline in the Int group. These levels changed to 22% and 16% insufficient at week 12, and to 44% and 26% insufficient in the Control group and Int groups, respectively, at week 52.

**FIG. 2.**
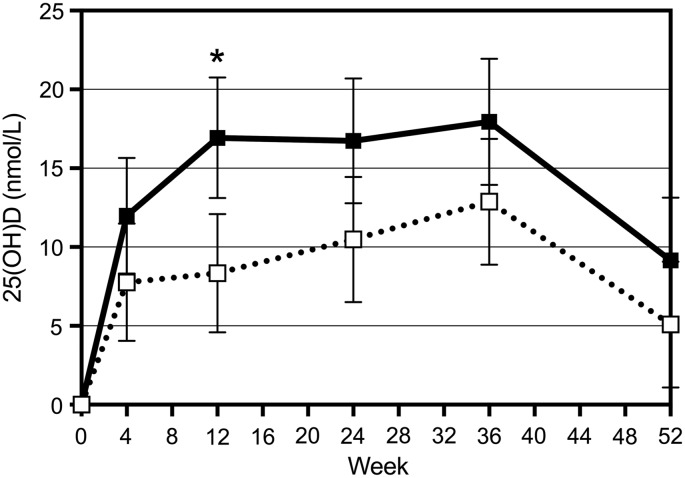
Change in 25(OH) vitamin D levels over 52 weeks of supplementation. ■, Int; □, Control. *p < 0.05 Int versus controls.

Mean serum 25(OH) vitamin D3 levels at baseline were insufficient (i.e., <50 nmol/L) for the Control and sufficient (>50 nmol/L) in the Int groups (Table 2). 25(OH) vitamin D3 levels increased from 48.6 to 55.0 in the Controls and from 53.2 to 60.8 nmol/L in the Int group. The change from baseline was significant for both groups (*p* = 0.013 for Control and *p* < 0.001 for Int), and at week 12, the levels for the Int group were significantly higher than the Controls (*p* = 0.003). Figure 2 shows the change from baseline over 52 weeks. In addition, the data indicated that for every extra 1 nmol 25(OH) vitamin D3 in serum at baseline, the change from baseline decreased by 0.63 nmol, suggesting that women with a higher vitamin D level at baseline responded less to the intervention.

#### Change in PTH

**FIG. 3.**
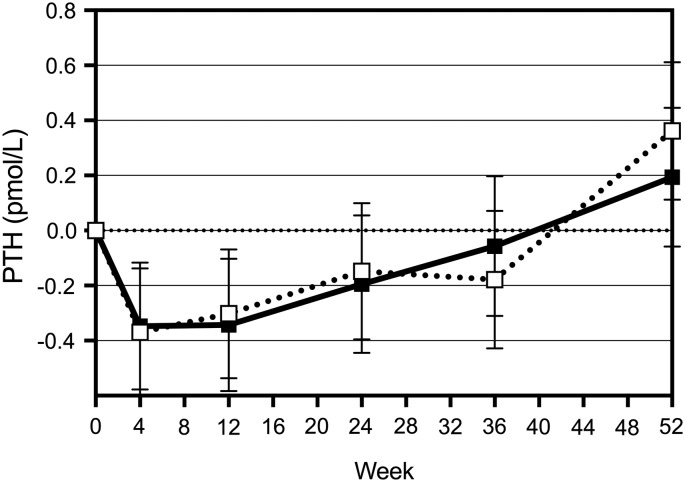
Change in PTH levels over 52 weeks of supplementation. ■, Int; □, Control. PTH, parathyroid hormone.

Figure 3 shows the change from baseline in PTH levels in the two groups of women over time. There was a significant time effect (*p* < 0.001) on PTH. Up until week 12, PTH decreased in both groups (*p* < 0.001), but then increased to a level slightly above the baseline value over time (*p* = 0.087 for week 0 vs. week 52).

#### Interaction between PTH and 25(OH) vitamin D3

**FIG. 4.**
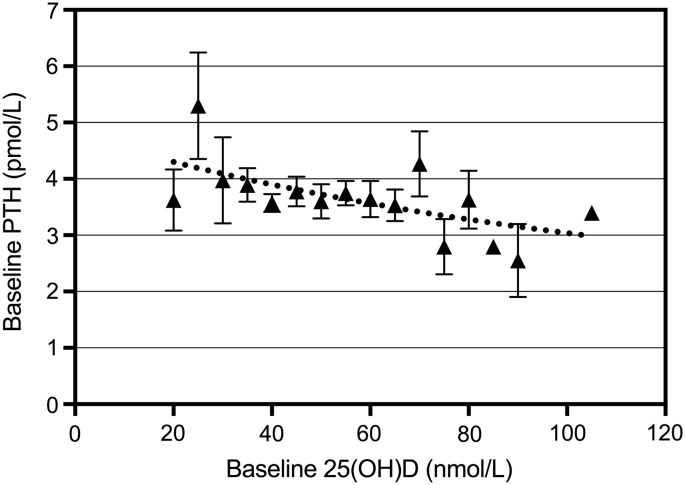
The relationship between baseline PTH and 25-hydroxyvitamin D [25(OH)D] (*r* = −0.14, *p* = 0.107 for linear relationship). 25(OH)D levels were grouped in intervals of 5 nmol/L. The curve represents the best fit to the data of one-phase exponential decay to a PTH plateau of 1.7 pmol/L (*r*^2^ = 0.38).

**FIG. 5.**
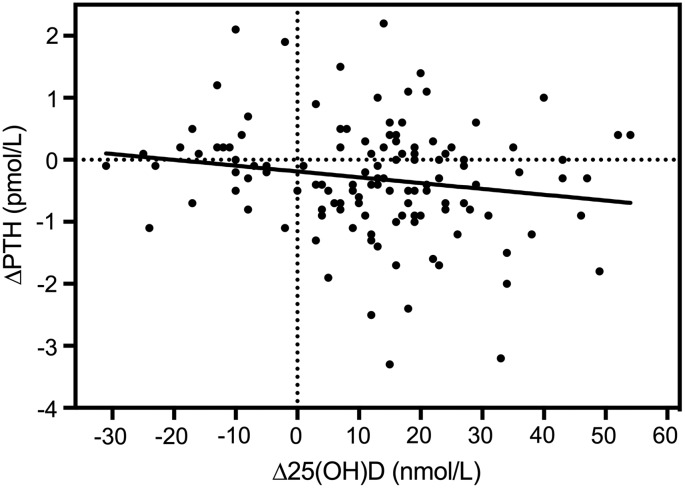
The relationship between changes in 25-hydroxyvitamin D [Δ25(OH)D] and ΔPTH after 12 weeks: *r* = −0.18, *p* = 0.052.

Baseline PTH and 25(OH) vitamin D3 levels were negatively correlated, with PTH showing a gradual decline with increasing 25(OH) vitamin D3. PTH seems to decline with levels of 25(OH) vitamin D3 up to 80 nmol/L. The relationship between baseline PTH and 25(OH) vitamin D3 is shown in Figure 4 (*r* = −0.14; *p* = 0.107). The 25(OH) vitamin D3 levels were grouped in intervals of 5 nmol/L, and the curve presents the best fit to the data of one-phase exponential decay, with PTH plateauing at 1.7 pmol/L (*r*^2^ = 0.38). Figure 5 shows the change in 25(OH) vitamin D3 and change in PTH over 12 weeks (*r* = −0.18; *p* = 0.052). This relationship remained negative for 12 weeks, but was not significant after 12 weeks due to a slow increase in PTH.

#### Change in CTx-1

CTx-1 levels for the Control as well as the Int group reduced within 4 weeks by 16.5 and 8.8%, respectively (*p* < 0.01). At week 12, the levels for both groups were at −7% and −10%, respectively, from baseline, and the levels remained stable until week 36. At week 52, both group's levels increased by 4–6%, but remained lower than baseline.

#### Changes in BMD

At baseline, body weight as well as whole body lean mass was positively associated with LS BMD (*p* = 0.006) and FN BMD (*p* = 0.051), however, fat mass was not associated with LS or FN BMD (*p* = 0.605 and *p* = 0.731). Whole body BMC increased in the Int group over the 52 weeks and this change was significant (*p* = 0.024). No effect of the supplementation was observed in the Control or the Int group over the 52 weeks for whole body, LS, or FN BMD (Table 4). FN Z-score remained unchanged in the Control group, but improved significantly in the Int group (*p* = 0.970 and *p* = 0.008 for difference from baseline; *p* = 0.051 for Int vs. Control).

**Table 4.** Lumbar Spine, Femoral Neck, and Whole Body Bone Mineral Content and Density at Baseline and at Week 52

Measurement	Control week 0	Control week 52	Int week 0	Int week 52
LS BMD	1.208 (0.0158)	1.214 (0.0156)	1.213 (0.0157)	1.219 (0.0155)
T-score	0.80 (0.132)	0.86 (0.130)	0.84 (0.131)	0.88 (0.129)
Z-score	0.69 (0.135)	0.71 (0.136)	0.63 (0.135)	0.72 (0.137)
FN BMD	0.893 (0.0176)	0.890 (0.0177)	0.869 (0.0185)	0.875 (0.0186)
T-score	−0.18 (0.147)	−0.20 (0.147)	−0.39 (0.154)	−0.33 (0.155)
Z-score	0.01 (0.140)	0.00 (0.141)	−0.31 (0.150)	−0.19 (0.150)^a^
WB BMC	2089 (29.4)	2089 (28.9)	2147 (29.2)	2153 (28.7)^a^
WB BMD	1.097 (0.0106)	1.101 (0.0112)	1.113 (0.0105)	1.120 (0.0111)
T-score	0.32 (0.094)	0.35 (0.098)	0.46 (0.093)	0.52 (0.097)
Z-score	0.27 (0.088)	0.28 (0.095)	0.36 (0.088)	0.43 (0.095)

Values are given as least square means (SEM). *N* = 28–67 per group.

^a^ Significantly different from baseline.

FN, femoral neck; WB, whole body.

#### Changes in body composition and grip strength

The Control group had an increase in whole body mass and lean mass, which was not detected in the Int group. There were no significant changes in fat percent in both groups over the 52 weeks. Grip strength (GS) between the groups was not significantly different at baseline (Table 2). However, GS was associated with body weight (*p* = 0.001) as well as whole body lean mass (*p* < 0.001) at baseline. GS increased over the 52 weeks in both groups of women with no significant difference between groups. However, women with a higher plasma 25(OH) vitamin D3 level at baseline tended to have a greater GS at baseline and at 24 weeks. Higher plasma 25(OH) vitamin D3 level at 12 weeks also tended to be associated with greater GS at 24 weeks. Furthermore, women with higher levels of plasma 25(OH) vitamin D3 or greater increases in plasma 25(OH) vitamin D3 levels during the first 4–24 weeks showed a greater increase in GS by week 24. Over the 52 weeks, the increase in GS was greater in the women who had a greater response to the supplemented vitamin D in the fortified milk.

## Discussion

In this study, we assessed the impact of a 52-week intervention with calcium fortified milk versus regular milk on general health, body composition, and bone density in premenopausal women. Both groups of women significantly improved their vitamin D status over the 52 weeks, with the Int group levels (who received 15 μg vitamin D in their fortified milk) remaining higher at all times compared to the Control group. At week 12, the Int group had significantly higher 25(OH) vitamin D3 levels than the Controls (*p* = 0.003) (Fig. 2). In both groups, CTx-1 as well as PTH levels were reduced by week 4 with no significant differences between groups over the 52 weeks (Fig. 3).

Even though baseline calcium intake was low, less than 500 mg/day, milk supplementation did not significantly affect BMD, with only slight increases in LS as well as FN BMD in both groups. In a small study, Baran et al.^8^ reported that vertebral bone density remained stable over 3 years of increased dairy food intake in women aged 30–42 years, while bone density declined in the control group and was significantly lower in the controls compared with the intervention group at the end of 3 years. A larger study in over 400 Chinese women aged 20–35 years (still increasing their peak bone mass), reported the effect of milk supplementation versus no supplement on bone density over 2 years and found that both groups had an increase in BMD, and at the end of the 24 months with no differences between groups. A possible lack of compliance was given as the reason for not seeing effects.^12^

In our study, over the 52 weeks, whole body BMC increased significantly in the Int group and FN Z-score improved significantly, which may indicate a positive effect on bone mineralization. The clinical goal of fracture risk management is to prevent the first fracture.^18^ The BMD of postmenopausal women is determined by bone acquired at the time of skeletal maturity (peak bone mass) and then menopausal- as well as age-related bone loss.^13^ The small but significant change in the femur BMD in the Int group may contribute to a reduction in fracture risk in future years.

Based on the IOM guidelines^17^ 44% of the women in the control group were vitamin D insufficient at week 52 with 26% being insufficient in the Int group. The change from baseline over the 52 weeks was significant for both groups (*p* < 0.001 compared to baseline), although the increase was greater in the Int group (*p* = 0.008 vs. control). There was also a significant effect of baseline 25(OH) vitamin D3 on the difference from baseline (*p* < 0.001); women with lower baseline vitamin D status responded better to the intervention.

A significant time effect on PTH levels was found in both groups and between weeks 24 and 52, the levels for both groups increased significantly (*p* = 0.004 and *p* = 0.0006, respectively). The latter increase could be due to reduction in compliance as the completed consumption diaries indicated a compliance that ranged between 86% and 82% over the 52 weeks. Bacon et al.^19^ reported a negative correlation between baseline PTH and 25(OH) vitamin D3 in premenopausal women, which is also shown for our cohort (Fig. 4). However, PTH declined up to 80 nmol/L of 25(OH) vitamin D3, whereas Bacon et al.^19^ reported no further change in PTH levels after a vitamin D status of 40 nmol/L. Both age and calcium intake affect the relationship between PTH and 25(OH) vitamin D3. Our cohort has similar baseline calcium intakes compared to the cohort discussed by Bacon et al.,^19^ but the relationship we found between PTH and 25(OH) vitamin D3 was not as strong. The baseline range of serum 25(OH) vitamin D3 levels for their population was 11 to 70 nmol/L, with at least 80% of the women being vitamin D insufficient, whereas about 60% of our cohort was insufficient at baseline. The latter may explain the weaker relationship recorded for our cohort. In our cohort, the change in PTH was related to the change in 25(OH) vitamin D3 levels (Fig. 5).

Our data indicate that body weight and lean mass at baseline, but not fat mass, are associated with LS BMD as well as FN BMD. Similar associations were also reported by Ho and Kung in 2005.^18^ Liu et al.^20^ investigated the relative contribution of lean and fat mass to bone density in young Chinese women. Fat mass was a major determinant for BMI, and BMI as well as lean mass was positively related to LS, total hip, and whole body BMD. The conclusion was that lean mass is an important factor determining BMD in young and premenopausal Chinese women. However, Fu et al. in 2011^21^ indicated that fat mass was positively associated with total and regional BMD (all *p* < 0.05). Yoo et al.^22^ reported that fat mass had a significant negative effect on bone mass in premenopausal women, but this was not observed in our study. Regression analysis of our data indicate that body weight can explain 8% of the variance in whole body (WB) BMD, while WB lean mass (LM) can explain 17% of the variance (*p* = 0.001). In addition, LM can explain 7% of the variance in LS BMD (*p* = 0.006).

GS as an indicator of muscle strength was also assessed over the 52 weeks of supplementation. Women with a higher 25(OH) vitamin D3 level at baseline tended to have a greater GS with change in GS up to week 52 correlating with change in vitamin D status over time. Von Hurst et al.^23^ reported that vitamin D status had a small but significant association with GS. Stewart et al.^24^ found that serum 25(OH) vitamin D3 levels was the common contributor to physical fitness indicators, including GS in postmenopausal women. In contrast, Goswami et al.^25^ found that calcium and vitamin D supplementation did not improve skeletal muscle strength in young females. In our study, vitamin D status was correlated with GS, and in addition, body weight and WB lean mass were also correlated with GS at baseline, and baseline lean mass explained 16.5% of the variation in GS. Sirola et al.^26^ reported that maintenance of GS is associated with reduced menopausal bone loss and reduced risk of future fractures. The small changes in GS that we observed may therefore be related to future bone health.

Our study had some limitations: multiple nutrients were included in the fortified milk and that resulted in a lack of ability to distinguish the specific effects of each; we did not have a nonsupplemented control group. Lappe and Heaney^27^ reviewed why randomized controlled trials of calcium and vitamin D often fail to show bone-specific effects even over more than 2 years' intervention. Critical criteria include the use of a single form of nutrient (due to the inclusion of calcium, vitamin D, and FOS-inulin, we cannot differentiate the single active component); the use of a low exposure control group (our controls also received milk) and the adequacy of the dose given to the treatment group. The baseline calcium intake of the women was 470 mg, but it could have blunted the response to both the control milk and the fortified milk.

We conclude that lean mass, and not fat mass, is the strongest predictor of BMD in our cohort of young women, and that improvement in vitamin D status observed in both groups affected GS dependent on baseline status and magnitude of improvement. We demonstrated a relationship between PTH levels and change in vitamin D status and a reduction in bone resorption in both groups. No significant changes were observed in LS or FN BMD, but the FN Z-score improved in the Int group. Milk supplementation resulted in positive effects on PTH and vitamin D status as well as bone health, with the fortified milk being measurably more effective.

## Abbreviations Used

BMD: bone mineral density
BMI: body mass index
CTx-1: C-telopeptide of type I collagen
FN: femoral neck
GS: grip strength
IOM: Institute of Medicine
LS: lumbar spine
PTH: parathyroid hormone
WB: whole body
